# Association between top-down skills and auditory processing tests

**DOI:** 10.5935/1808-8694.20130137

**Published:** 2015-10-08

**Authors:** Cristina Ferraz Borges Murphy, Renata La Torre, Eliane Schochat

**Affiliations:** a Post-Doctorate Student, University of São Paulo; Speech and Hearing therapist; b Degree in Speech and Hearing Therapy, University of São Paulo; c Senior Associate Professor, University of São Paulo. University of São Paulo

**Keywords:** attention, auditory perception, hearing tests, language, memory

## Abstract

Today, we are questioning how top-down skills may interfere with performance on auditory processing tests.

**Objective:**

To investigate the existence of a possible association between memory, attention and language skills in auditory processing tests in “*normal*” development children.

**Method:**

Twenty children (ages 7 to 9 years), without complaints related to verbal and/or written language skills; without overt neurological or psychological involvement or delayed psychomotor development. We employed Hearing and auditory Processing Assessment tests in addition to psychophysical tests (visual and auditory attention tests; memory tests for digits and syllables and phonological awareness tests).

**Results:**

there was a “very strong” correlation between Frequency Pattern and Memory for Digits Tests; a “strong” correlations between SSW (LE) test and Memory for Syllables, and SSW (LE) test and phonemic tasks.

**Conclusion:**

the Frequency Pattern Test showed a strong correlation with the phonological working memory skill; just as the SSW had with language and memory skills for syllables. It is noteworthy the difficult to clinically interpret the results of each auditory processing test alone, since these may be dependent on skills not necessarily related to the auditory modality, such as memory and language.

## INTRODUCTION

Many studies have shown that the Auditory Processing Disorder (APD) usually happens concurrently with other disorders such as Attention Deficit and Hyperactivity Disorder (ADHD)1., 2., 3. and dyslexia4., 5., 6., 7.. However, it is still controversial whether these disorders are interdependent or just comorbidities3., 8., 9., 10., 11.. Some of the factors associated with this issue are the characteristics of the auditory processing tests applied, or rather what is genuinely assessed in each test. It is discussed how these are to be interpreted and how non-sensory skills - not necessarily related to hearing - may influence results and hence APD diagnosis^11^.

Numerous studies have reported the presence of APD in children with ADHD based on the observation of their poor performance in these auditory processing tests1., 2., 3.. Nevertheless, it is discussed whether this poor performance would not just be a secondary phenomenon to their lack of attention, for there is a strong influence of attention skills in these auditory processing tests2., 3., 4., 5., 6., 7., 8., 9., 10., 11., 12.. For instance, some authors have associated the high degree of response variability in some auditory processing tests, such as auditory temporal tests and attention skills, demonstrating the influence of this top-down skill^12^. Bellis et al.^10^ reported that the assessment of auditory processing is able to pinpoint children with ADHD - since these groups differ mainly on intra tests performance.

As far as dyslexia is concerned, since the 90s, studies corroborate the hypothesis initially advocated by Tallal that reading disorders are related to a change in auditory temporal processing4., 5., 6., 7.. Nevertheless, there is still controversy about this association, due to the difficulty in establishing a causal relationship between the two disorders, plus a large individual variability in the performance of these children vis-à -vis the temporal tests4., 5., 6., 7., 8., 13..

According to Salles^14^, reading can be considered a complex activity made up of a series of interdependent cognitive processes such as memory, attention, automatism, and phonological processes. We then discuss the common factors related to these processes involved in reading and, at the same time, involved in auditory processing skills. Is it possible that the results of auditory processing tests are indirectly influenced by these other skills, which are also indirectly evaluated on reading tests such as phonological awareness?

Based on the aforementioned studies, we may conclude that there is no consensus on how performance on auditory processing tests can be influenced by more overarching skills, such as attention, memory and language. Moreover, to date we do not know just how each of these non-sensory skills may impact performance on each auditory processing test.

To clarify these issues as to the feasibility of auditory processing tests in assessing non-sensory skills, this study aims to investigate a possible association of memory, attention and language skills and auditory processing tests in children considered with “typical” development. The hypothesis is that there is a specific correlation between the skills tested and certain auditory processing tests applied.

## METHOD

The study was approved by the Ethics Committee of the institution where it was executed, under research protocol # 575/09.

We had a single group with 20 children taking part in this study. We recruited the subjects from two elementary schools. The selection was made primarily by the teachers, using the criteria of age, gender and having no reading complaints (considering the student's academic performance). The subjects attended the Audiology Service of the institution responsible for the study, and they were submitted to the procedures listed below:

• Receiving and signing the Informed Consent Form;
• Interview, to investigate compliance with the study's inclusion criteria: age between 7 and 9 years and 11 months; both genders; no complaints related to overt reading, neurological or psychological disorder; no delayed psychomotor development or delays in the acquisition of oral language; no past of otitis and musical knowledge - i.e. knowing how to play a musical instrument or be in the process of learning to;
• Basic Audiological Evaluation (audiometry, speech audiometry and impedance test) to investigate the criterion: “Hearing within normal limits”. Individuals who did not have results within the normal range, according to the ANSI 69 standard, were taken off the study and referred to the specialist;
• Auditory Processing Assessment: Speech with Noise^15^, PSI^15^, SSW^15^, Nonverbal Dichotic^15^, Frequency Pattern^16^, GIN^17^. The monotic tests (Speech in Noise and PSI) were performed at an intensity of 40 dB SL in relation to the Speech Recognition Threshold (SRT) value: the dichotic (SSW and Dichotic Nonverbal) and temporal (Frequency Pattern and GIN) tests were performed at an intensity of 50 dB SL to the SRT value. For the Speech in Noise test, we used the signal/noise ratio of -15 dB SL and, for the PSI test (speech/speech), we used the signal/noise ratio of -15 dB SL. In addition, we considered the specific normal standards for each test and age range (Speech with Noise^15^, PSI^18^, SSW^19^, Nonverbal Dichotic^15^, Frequency Pattern^20^, GIN^21^). The choice of these tests is related to the hearing skill investigated in each one of them (auditory closure, figure-background, binaural integration, ordering and temporal resolution), considered essential for AP assessment^15^.

### Psychophysical tests

#### Attention Tests

• Visual Attention Test. The test was developed using the E-Prime Professional Software and the Posner^22^ model. This model is widely described in the literature and can be considered as a standard criterion to investigate sustained attention. Numbers from one to seven are visually presented on the computer screen for four minutes. The children are then instructed to press the “space bar” only when the numbers one and five are shown. We count a total of 140 attempts. An error is computed when the key is not pressed for numbers one and five and when it is pressed for the remaining numbers;
• Test of Auditory Attention. Just like the Visual Attention test, this test has also been developed using the E-Prime Professional Software, with the Posner^22^ model. Numbers from one to seven are presented out loud during four minutes for the investigation of sustained attention. The children are instructed to press the “space bar” just for numbers one and five. They have a total of 140 attempts. An error is considered when they do not “press the space bar” for the numbers one and five and when they do it for the remaining numbers.

#### Memory Test

• Memory test for digits (Digit Span) developed and implemented from the E-Prime Professional Software. According to the initial model proposed by Baddeley & Hitch^23^ the Digit Span test enables the investigation of phonological working memory. The Span task starts with sets of three digits and 12 trials for each series. Children are instructed to verbally repeat the sequence of numbers in direct order after each attempt is shown on the computer screen. If the child scores over 50%, i.e. more than six correct attempts at each series incrementally, series with more digits will be presented. The last series in which there was more than 50% accuracy is considered as the Span result;
• Memory Test for Syllables (Syllable span) - developed and implemented using the E-Prime Professional Software. It enables the assessment of phonological working memory, also following the model proposed by Baddeley & Hitch^23^. The syllables that make up the test have plosive and fricative consonants in the initial position (/ba/,/bo/,/da/,/de/,/di/,/pa/,/co/,/fa/,/fe/,/fi/,/cha/,/chi/,/cho/,/ga/) and they were recorded in the Cinema, Radio and Television Studio of the School of Communication and Arts, University of São Paulo (ECA- USP). The Span task starts with sets of three syllables presented verbally, 12 trials for each series. Children are instructed to repeat a sequence of syllables in direct order after hearing each attempt. If the child performs better than 50%, i.e. more than six correct attempts, series with more syllables are gradually introduced. The last series with more than 50 % accuracy is considered the Span result.

#### Language Test

• Phonological Awareness Test - adapted from the Phonological Awareness Test - PCF^24^. This test contains 10 tasks: syllabic and phonemic synthesis; syllabic and phonemic segmentation; rhyme; alliteration; syllabic and phonemic manipulation, syllabic and phonemic transposition. Each task has five items corresponding to the test.

#### Statistical Analysis

To investigate whether there is correlation between performances in each test, we employed the Spearman correlation with significance set at *p* < 0.05. The degree of correlation was established from the values described on Table 1^25^.

**Table 1:** Correlation degree^25^.

Coefficient	Correlation
0-0.25	Very weak
0.25–0.50	Weak
0.5–0.75	Moderate
0.75–0.9	Strong
0.9–1	Very Strong

## RESULTS

Prior to the analysis of the correlation between the performances in each test, the group profile will be characterized from the data on Table 2.

**Table 2:** Mean score and standard deviation for each test.

Group		
Gender, boys/girls	14/6	
Age, years	8.2 ± 0.76	
Auditory Processing	RE	LE
PSI (total 10)	8 ± 2.31	7.65 ± 1.22
Speech with Noise (total 25)	17.85 ± 2.66	18.75 ± 2.73
Nonverbal Dichotic (total 12)	9.4 ± 2.74	10 ± 2.94
SSW (total 40)	31 ± 6.22	29 ± 9
Frequency Pattern (total 20)	15.1 ± 4.17	
GIN	4.3 ± 0.48	
Memory		
Digits Span	5.35 ± 1.13	
Syllables Span	4.8 ± 1.05	
Attention		
Visual Attention (total 210)	200.75 ± 11.56	
Hearing Attention (total 210)	190.25 ± 11.63	
Phonological Awareness		
Syllable tasks (total 16)	15.8 ± 0.52	
Phonemic tasks (total 16)	7.8 ± 4.74	
Rhyme and alliteration (total 8)	7 ± 1.8	

PSI: Pediatric Speech Intelligibility; SSW: Staggered Spondaic Word; GIN: Gap in Noise.

Table 2 depicts the group profile in terms of age, gender and performance in each of the tests. The averages are in percent values, except for the GIN, Digit and Syllables Span tests. In the phonological awareness test, we decided to group the performances obtained in the phoneme (synthesis, phonemic and manipulation segmentation), syllables (synthesis, segmentation and syllabic manipulation) and rhyme and alliteration to facilitate analysis.

In auditory processing tests, the group performance was within what was expected for all tests, except for the Nonverbal Dichotic with underperformance for the children in the age groups studied (expected pattern of 11 hits for each ear with guided attention; the group got 9.4 correct answers in the right ear and 10 in the left ear). The other tests employed (memory, attention and language) did not exhibit the normality pattern established.

Table 3 shows the correlation between each auditory processing test and the Attention (Visual and Auditory Attention); Memory (Digit Span and Syllables Span) and Language (phonological awareness) tests.

**Table 3:** Correlation between performance on auditory processing, attention and language tests.

		Visualattention	Auditoryattention	DigitsSpan	SyllablesSpan	Rhyme and Alliteration	Syllabletasks	Phonemictasks
	Coefficient	0.321	0.083	0.223	0.244	0.141	-0.061	0.234
PSI_RE	^*p*^	0.168	0.727	0.346	0.299	0.553	0.797	0.322
	N	20	20	20	20	20	20	20
	Coefficient	0.122	0.045	0.552	0.558	0.517	0.415	0.416
PSI_LE	^*p*^	0.607	0.852	0.012^*^	0.011^*^	0.019^*^	0.069	0.068
	N	20	20	20	20	20	20	20
	Coefficient	0.422	-0.004	0.233	0.129	-0.096	-0.025	0.266
SN_RE	^*p*^	0.064	0.986	0.322	0.587	0.687	0.918	0.258
	N	20	20	20	20	20	20	20
	Coefficient	0.296	0.017	0.306	0.117	0.122	-0.026	0.297
SN_LE	^*p*^	0.204	0.943	0.189	0.623	0.607	0.913	0.204
	N	20	20	20	20	20	20	20
	Coefficient	0.448	0.549	0.589	0.492	0.412	0.262	0.490
DNV_RE	^*p*^	0.048^*^	0.012^*^	0.006^*^	0.027^*^	0.071	0.264	0.028^*^
	N	20	20	20	20	20	20	20
	Coefficient	0.435	0.578	0.736	0.546	0.479	0.500	0.648
DNV_LE	^*p*^	0.055	0.008^*^	0.000^*^	0.013^*^	0.032^*^	0.025^*^	0.002^*^
	N	20	20	20	20	20	20	20
	Coefficient	0.574	0.451	0.739	0.612	0.505	0.327	0.680
SSW_RE	^*p*^	0.008^*^	0.046^*^	0.000^*^	0.004^*^	0.023^*^	0.159	0.001^*^
	N	20	20	20	20	20	20	20
	Coefficient	0.379	0.298	0.652	0.805	0.533	0.511	0.865
SSW_LE	^*p*^	0.100	0.202	0.002^*^	0.000^*^	0.016^*^	0.021^*^	0.000^*^
	N	20	20	20	20	20	20	20
	Coefficient	0.516	0.205	0.929	0.705	0.489	0.253	0.635
FP	^*p*^	0.049^*^	0.464	0.000^*^	0.003^*^	0.065	0.363	0.011^*^
	N	15	15	15	15	15	15	15
	Coefficient	0.022	0.201	0.346	-0.047	0.049	0.284	0.159
GIN	^*p*^	0.942	0.511	0.247	0.879	0.873	0.347	0.605
	N	13	13	13	13	13	13	13

PSI: Pediatric Speech Intelligibility; SN: Speech in Noise; DNV: Dichotic nonverbal; SSW: Staggered Spondaic Word; FP: Frequency Pattern; GIN: Gap in Noise; p: Significant value; N: Number of participants; RE: Right ear, LE: Left ear; ^*^ Significant.

The significant correlations considered “strong” or “very strong” were highlighted in bold letters. They are: Frequency Pattern and Digits Span (“strong”) tests; SSW (LE) and Memory for Syllables (“strong”) tests and SSW (LE) test and phonemic tasks (“strong”).

## DISCUSSION

The group showed average performance below expectations in the Nonverbal Dichotic test. Perhaps the fact that we included children from public schools, i.e. children from a low socioeconomic level, might have favored the inclusion of children without the stimulation needed for the development of certain hearing skills, as was the case in this particular skill. Nevertheless, underperformance is used as a criterion to diagnose CPAD, substandard performance (two standard deviations or more) in at least two auditory processing tests26., 27.. Thus, a change in a hearing skill alone, as it happened in this study, does not constitute a diagnosis of Auditory Processing Disorder; moreover, in most cases, it is not enough for the patient to have a hearing complaint, which would explain their inclusion in the study even after the medical interview is carried out with the parents.

Table 3 depicts the correlations between each Auditory Processing test and attention, memory and language skills.

The SSW (LE) test presented a correlation deemed “strong” with the memory for Syllables Tests and Phonological Tasks.

The Memory for Syllables Test analyzes the phonological working memory. This memory is related to the storage or retention of unfamiliar sound patterns at the time when a record is built in a more permanent memory; also, secondarily, it retains sequences of familiar words^28^. This sequencing of words is one of the skills assessed in the SSW test, since the test's goal is to repeat a sequence of four words heard. Therefore, this corresponds to an aspect in common between the two tests, which could explain the correlation.

Tests involving phonemic tasks are tests that assess the phonological awareness skill. This ability refers to both: the awareness that speech can be segmented as well as the ability to manipulate such segments^29^. By means of the dichotic listening task, the SSW test analyzes the ability of the individual to identify overlapping words. Thus, considering the characteristics of each test, it is assumed that a good performance in both depends on skills related to the auditory perception of phonemes - a factor that could explain the high correlation found. Furthermore, it is important to mention that the strongest correlations were found only for the left ear. This result may be related to the left-hemisphere dominance for speech and language processing and dichotic listening^30^. It is known that in dichotic listening tests the contralateral pathway is largely responsible for the processing of information. Thus, for the left ear, one needs a longer processing time, since after the information gets to the right hemisphere, it must cross to the opposite hemisphere through the corpus callosum. Perhaps, this longer processing time for the left ear is responsible for better showing the influence of phonological awareness in this ear.

The Frequency Pattern Test had a deemed “very strong” correlation with the Memory for Digits test, responsible for working memory analysis. According to Baddley & Hitch^23^, the main working memory component is the central executive system, which has attention resources that enable the execution of concurrent tasks needed in different problem situations, such as mathematical problem solving, text reading comprehension, etc. In the current study, the Frequency Pattern Test was applied by means of a verbal response. This type of response requires the individual to memorize the association between the name (low or high) and the specific sound, ensuring a correct naming of the sound, while memorizing the sequence of sounds heard in order to sort the stimuli in their order of appearance. The hypothesis is that perhaps the concurrent execution of these tasks involves the working memory, which could explain the correlation. Moreover, if we consider the relationship between working memory and reading tasks such as reading comprehension, we could suggest that perhaps the subgroup of children with dyslexia who had alterations in this type of memory is more prone to exhibit poor performance in tests like this.

The psychophysical attention tests were only moderately correlated with some AP tests (Nonverbal Dichotic, right ear SSW and Frequency Pattern). Two hypotheses are considered: the first is that maybe these tests are not sensitive enough to detect any major variation in performance for this skill. Note, for instance, that the mean score was higher than 90% for both tests (Table 2) and with a small standard deviation. The second hypothesis is that perhaps the performance on auditory processing tests is not directly related to performance on attention tests. This hypothesis would corroborate the results from Bellis's intra-tests, who claimed he did not find similar profiles between groups of children with auditory processing disorders and ADHD groups in auditory processing tests.

## CONCLUSION

The results of this study show a correlation between performance on certain auditory processing (Frequency Pattern and SSW) tests and certain skills considered “top down” (memory and language). This result highlights the difficulty in clinically interpreting the results of each auditory processing test alone, since these may be dependent on certain skills not necessarily associated with hearing. Therefore, we stress the importance of a multidisciplinary approach aimed at finding differential diagnosis among disorders with similar profiles such as Learning Disorders and Auditory Processing Disorder.
